# Editorial: Growth and Tissue Regeneration with focus on Proteoglycans

**DOI:** 10.3389/fcell.2023.1139044

**Published:** 2023-03-13

**Authors:** Mona Elisabeth Pedersen, Sissel Beate Rønning, Svein Olav Kolset

**Affiliations:** ^1^ Nofima AS-Norwegian Institute of Food, Fisheries and Aquaculture Research, Tromsø, Norway; ^2^ Institute of Basic Medical Sciences, Faculty of Medicine, University of Oslo, Oslo, Norway

**Keywords:** proteoglycans, tissue regeneration, remodeling, signaling, shedding

A relevant aim in PG biology is to identify molecurlar mechanisms on how PG modulates cell growth and regeneration processes, aiming at possibilities for future use as therapeutic targets. Examples of PG relevance within the medical field include cardiac hypertrophy, wound healing, angiogenesis, and hair follicle renewal processes. A striking finding in this Research Topic was presented in the work of Parsberg Støle et al., showing that there are *significant sex differences* in the molecular mechanisms driving the adaptive remodeling of the cardiac heart in response to biomechanical stress. Medical findings are often based on pre-clinical studies in male mice, missing out on crucial information for medical treatment for females. In response to long-term biomechanical stress, extensive cardiac remodeling, and hypertrophy, resulting in fibrosis and heart failure, involve PGs. The ubiquitously expressed proteoglycan syndecan-4 is involved in developing hypertrophy in the pressure-overloaded heart. Parsberg Støle et al.'s paper compared female and male WT hearts with syndecan-4 KO mice. Compared to female WT hearts, female syndecan-4 KO cardiomyocytes were smaller. Furthermore, these hearts had higher levels of pSer473-Akt and its downstream target pSer9-GSK-3β. The pSer473-Akt inhibitory phosphatase PHLPP1/SCOP was lowered, which may be in response to the elevated serum insulin levels found in the female syndecan-4 KO mice. In contrast, in male syndecan-4 KO mice, the pSer473-Akt levels were unaltered compared to WT. Syndecan-4 is a relevant target in studies of sex differences and heart failure. The basement proteoglycan perlecan is another example of a ubiquitous, multifunctional, and pleomorphic molecule with potential as therapeutic target, reviewed by Melrose et al. *Perlecan domain V promotes tissue repair* through interactions with VEGF, VEGF-R2, and α2β1 integrin and perlecan domain-V LG1-LG2 and LG3 fragments antagonize these interactions. Further, perlecan domain V promotes reconstitution of the blood-brain barrier damaged by an ischemic stroke. Perlecan interactions with different growth factors promote angiogenesis and wound healing, which is relevant for developmental and repair processes. Glypicans (GPCs), are also interesting therapeutic targets for repair processes. In alopecia, the hair cycle is accelerated, resulting in thinner and shorter hair formation. In addition, alopecia is associated with a decrease in the micro-vascularization of the hair follicles. In the work of Brézillon et al., the role of glypicans (GPCs) in the angiogenesis of human dermal microvascular endothelial cells was analyzed. The HDMEC pseudotube formation was shown to be abrogated after GPC1 siRNA transfection of HDMEC. GPC1 also interacts directly with VEGFR2 or c-Met to regulate angiogenesis. Altogether, these results showed that *GPC1 might constitute a potential target to influence alopecia* in dermatology research. PGs and their interactions *via* GAG chains or shedding of the extracellular part of cell surface PGs are also potential therapeutic targets for tissue homeostasis in repair biology. About 30%–50% of renal allografts are lost after 5 years of transplantation. Chronic transplant dysfunction (CTD) is associated with increased PCSK9 and dyslipidemia. Syndecan-1 act as a co-receptor for PCSK9 and shedding of syndecan-1 has been associated with increased serum triglyceride levels after renal transplantation. Treatment with negatively charged heparin (oids) blocking PCSK has been suggested as a therapeutic option to improve dyslipidemia and CTD. Van den Born et al. demonstrate in their paper that the *efficacy of heparin and non-anticoagulant heparins in lipid reduction is controversial and context-dependent*. Based on their results, the authors conclude that relying entirely on heparin and non-anticoagulant heparins in preventing CTD and CTD-related tissue remodeling might not be warranted, at least not in the kidney transplantation setting.

## Concluding remarks

The articles collected in this Research Topic demonstrate the complexity of PGs, the diversity of their biological functions, depending on tissues studied and diseases used as basis for functional studies. The papers presented address important functional aspects of some distinct PGs, and provide basis for more studies in different diseases.

